# Red/green cyanobacteriochromes acquire isomerization from phycocyanobilin to phycoviolobilin

**DOI:** 10.1002/pro.5132

**Published:** 2024-07-29

**Authors:** Hiroki Hoshino, Keita Miyake, Keiji Fushimi, Rei Narikawa

**Affiliations:** ^1^ Graduate School of Science Tokyo Metropolitan University Hachioji Tokyo Japan; ^2^ Graduate School of Arts and Sciences University of Tokyo Meguro Tokyo Japan

**Keywords:** bilin, cyanobacteriochrome, molecular evolution, phytochrome

## Abstract

Cyanobacteriochromes (CBCRs) are unique cyanobacteria‐specific photoreceptors that share a distant relation with phytochromes. Most CBCRs contain conserved cysteine residues known as canonical Cys, while some CBCRs have additional cysteine residues called second Cys within the DXCF motif, leading to their classification as DXCF CBCRs. They typically undergo a process where they incorporate phycocyanobilin (PCB) and subsequently isomerize it to phycoviolobilin (PVB). Conversely, CBCRs with conserved Trp residues and without the second Cys are called extended red/green (XRG) CBCRs. Typical XRG CBCRs bind PCB without undergoing PCB‐to‐PVB isomerization, displaying red/green reversible photoconversion, and there are also atypical CBCRs that exhibit diverse photoconversions. We discovered novel XRG CBCRs with Cys residue instead of the conserved Trp residue. These novel XRG CBCRs exhibited the ability to isomerize PCB to PVB, displaying green/teal reversible photoconversion. Through sequence‐ and structure‐based comparisons coupled with mutagenesis experiments, we identified three amino acid residues, including the Cys residue, crucial for facilitating PCB‐to‐PVB isomerization. This research expands our understanding of the diversity of XRG CBCRs, highlighting the remarkable molecular plasticity of CBCRs.

## 1 INTRODUCTION

Photosynthetic organisms rely on photoreceptors to perceive their surrounding light conditions, enabling efficient photosynthesis. Among these receptors, phytochromes, which are linear tetrapyrrole‐binding photoreceptors, are vital in various light‐related processes in land plants, including shade avoidance response, germination, and light acclimation (Fushimi & Narikawa, 2021a; Rockwell & Lagarias, 2010). Distinct from phytochromes, cyanobacteriochromes (CBCRs) are also linear tetrapyrrole‐binding photoreceptors, distantly related, and are specific to cyanobacteria (Fushimi & Narikawa, 2019; Fushimi & Narikawa, 2021a). CBCRs play roles in diverse light acclimation processes such as phototaxis and chromatic acclimation (Enomoto et al., 2014, 2015; Hirose et al., 2010; Kehoe & Grossman, 1996; Narikawa et al., 2011; Song et al., 2011; Wiltbank & Kehoe, 2016; Yoshihara et al., 2000). Both types of photoreceptors undergo reversible photoconversion between two light‐absorbing states and demonstrate *Z*/*E* isomerization of the C15 = C16 double bond of the chromophore as a primary photoreaction (Figure S1A). Notably, CBCRs require only the cGMP‐phosphoesterase/adenylate cyclase/FhlA (GAF) domain for chromophore binding and light sensing. In contrast, phytochromes require Per‐Arnt‐Sim, GAF, and phytochrome‐specific domains in most cases.

The majority of CBCR GAF domains feature conserved cysteine residues, known as canonical Cys, which establish a stable covalent bond to C3^1^ (Rockwell & Lagarias, 2010) of the chromophore (Figure S1B, C) (Fushimi & Narikawa, 2019). Additionally, some CBCR GAF domains contain an extra cysteine residue within the Asp‐Xaa‐Cys‐Phe (DXCF) motif, called second Cys. This second Cys is involved in chromophore isomerization from phycocyanobilin (PCB) to phycoviolobilin (PVB) and forms a covalent bond with the C10 of the chromophore during photoconversion (Figure S1B). This results in a significant blue shift due to shortened conjugated system (Figure S1B) (Burgie et al., 2013; Cho et al., 2015; Enomoto et al., 2012; Hasegawa et al., 2018; Ishizuka et al., 2006, 2007; Ma et al., 2012; Narikawa et al., 2011, 2013; Rockwell et al., 2008; Rockwell, Martin, Gulevich, & Lagarias, 2012; Rockwell, Martin, & Lagarias, 2012b; Song et al., 2011; Yoshihara et al., 2004). These CBCR GAF domains, possessing both canonical and second Cys, are termed DXCF CBCR GAF domains. Typical DXCF CBCR GAF domains bind PVB and demonstrate reversible photoconversion between a blue‐absorbing (Pb) dark‐adapted state, binding the *Z*‐configurated PVB and a green‐absorbing (Pg) photoproduct state, binding the *E*‐configurated PVB (Figure S1B). The presence of DXCF CBCR GAF domains has been observed across various lineages and is believed to have originated early in CBCR evolution (Rockwell & Lagarias, 2023).

**FIGURE 1:**
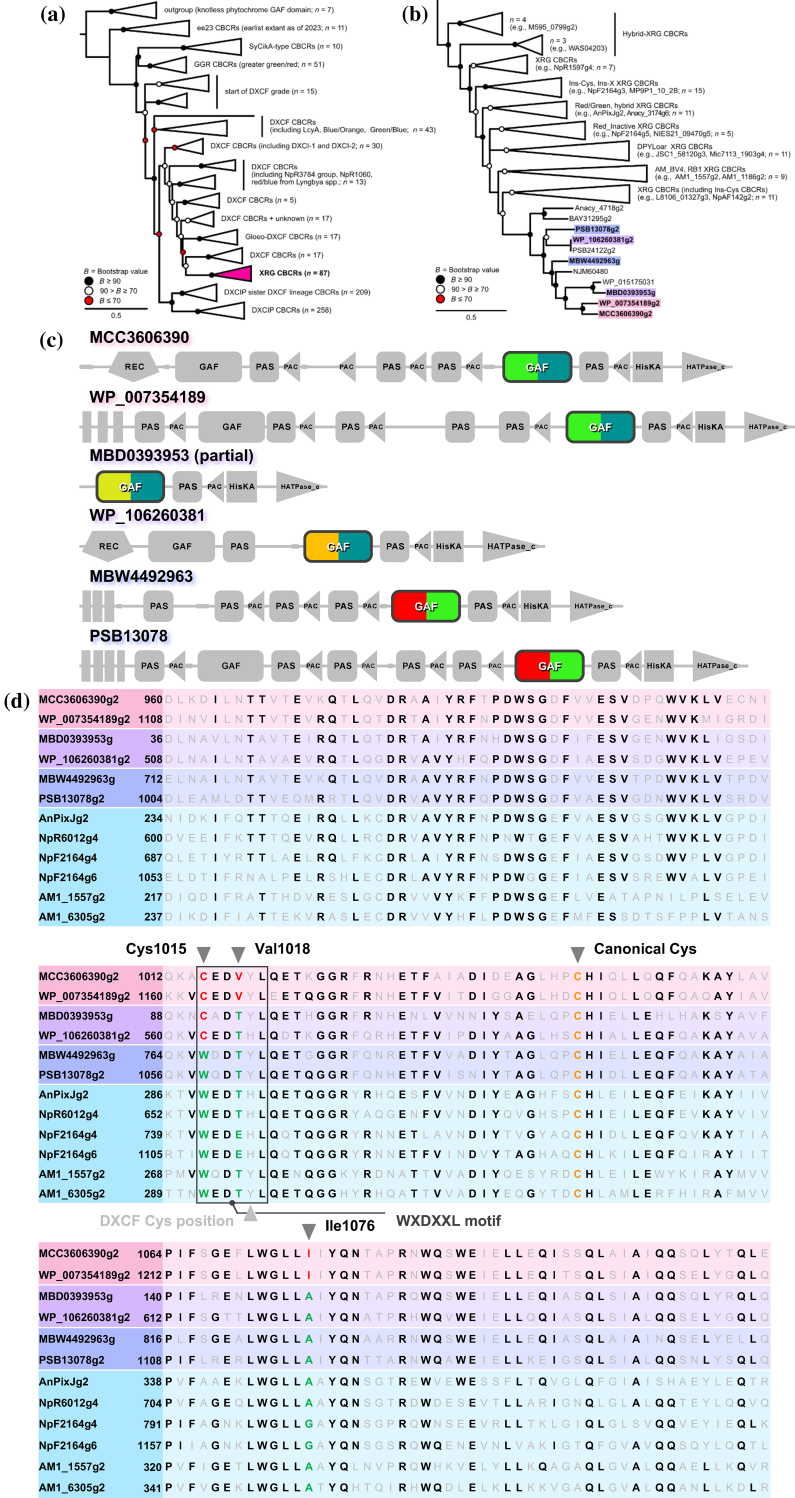
Phylogenetic analysis, domain architecture, and multiple sequence alignment of novel XRG CBCRs. (a) Phylogenetic tree depicting representative CBCR GAF domains. XRG CBCRs are highlighted in red. (b) Phylogenetic tree specifically focusing on XRG CBCR GAF domains. Novel XRG CBCR GAF domains from this study are denoted in pink (MCC3606390g2 and WP_007354189g2), purple (MBD0393953g and WP_106260381g2), and blue (MBW4492963g and PSB13078g2). (c) Domain architecture of novel CBCRs, with GAF domains colored based on the absorbed light colors in the dark‐adapted state and photoproduct state. (d) Multiple sequence alignment of XRG CBCR GAF domains, with the first amino acid number listed on the left. Conserved amino acid residues are highlighted in bold and the three key amino acid residues are colored in red.

The CBCR GAF domains with the highly conserved Trp‐Xaa‐Asp‐Xaa‐Xaa‐Leu (WXDXXL) motif are known as extended red/green (XRG) CBCR GAF domains (Chen et al., 2012; Narikawa et al., 2008; Rockwell, Martin, & Lagarias, 2012c). The XRG lineage emerged from the DXCF lineages by losing the DXCF second Cys residue in a later phase of CBCR evolution (Figure 1a) (Rockwell & Lagarias, 2023). Typical XRG CBCR GAF domains bind PCB and undergo reversible photoconversion between a red‐absorbing (Pr) dark‐adapted state binding the *Z*‐configured PCB and a Pg photoproduct state binding the *E*‐configured PCB. These typical GAF domains are called red/green CBCR GAF domains, the narrow definition of part of the XRG CBCR GAF domains. These red/green CBCR GAF domains show an interaction between the Trp residue on the WXDXXL motif and the pyrrole rings of PCB (i.e., hydrogen bond with the A‐ring and π–π stacking with the D‐ring of the chromophore) (Figure S1C) (Chen et al., 2012; Narikawa et al., 2008; Rockwell, Martin, & Lagarias, 2012c). XRG CBCR GAF domains are highly diversified, with some acquiring insertion sequences (Rockwell et al., 2022; Rockwell, Martin, & Lagarias, 2012c), others losing photoconversion ability and specializing in fluorescence (Fushimi et al., 2016, 2019; Narikawa et al., 2015), and still others gaining biliverdin‐binding ability (Fushimi et al., 2016, 2019; Narikawa et al., 2015). Moreover, CBCR GAF domains within the XRG lineage that reacquire the DXCF second Cys have been identified (Rockwell, Martin, & Lagarias, 2017). These CBCR GAF domains with the DXCF motif demonstrate both PCB‐to‐PVB isomerization ability and reversible ligation ability of C10 of the chromophore. In this context, the XRG CBCR GAF domains include not only typical red/green CBCR GAF domains but also these diversified CBCR GAF domains. These findings indicate that the XRG CBCR GAF domains exhibit remarkable plasticity.

Here, we report novel XRG CBCR GAF domains featuring the Cys‐Xaa‐Asp‐Xaa‐Xaa‐Leu (CXDXXL) motif instead of the conserved WXDXXL motif. These XRG CBCR GAF domains, lacking the DXCF second Cys, exhibited PCB‐to‐PVB isomerization ability similar to DXCF CBCR GAF domains. They exhibited reversible photoconversion between a Pg dark‐adapted state and a teal‐absorbing (Pt) photoproduct state but without reversible Cys ligation to the C10 of the chromophore. Through sequence‐ and structure‐based comparisons, we identified three amino acid residues crucial for PCB‐to‐PVB isomerization activity. This study unravels the diversity of XRG CBCR GAF domains and highlights the remarkable plasticity in the molecular evolution of CBCRs.

## 2 RESULTS

### 2.1 Discovery of novel XRG CBCR GAF domains lacking a conserved tryptophan residue in the WXDXXL motif

CBCRs have expanded their diversity through the acquisition of Cys residues involved in chromophore structural isomerization and/or covalent bond formation with the chromophore during photoconversion (Blain‐Hartung et al., 2021; Narikawa et al., 2014; Rockwell et al., 2011, 2017; Rockwell & Lagarias, 2023). We anticipated the discovery of novel CBCRs with unique characteristics due to the acquisition of Cys residues near the chromophore. To explore this possibility, we conducted a search for novel CBCR GAF domains containing Cys residues near the chromophore using protein BLAST. As a result, we identified uncharacterized XRG CBCR GAF domains, which formed a distinct cluster within the XRG lineage (Figure 1b). Among them, four GAF domains, MCC3606390g2 from *Microcoleus* sp. PH2017_29_MFU_D_A, WP_007354189g2 from multispecies *Kamptonema*, MBD0393953g from *Microcoleus* sp. C1‐bin4, and WP_106260381g2 from *Stenomitos frigidus*, have the CXDXXL motif instead of the WXDXXL motif, which is highly conserved in the XRG lineage (Figure 1c, d). In contrast, the other two homologous GAF domains, MBW4492963g from *Oscillatoria princeps* RMCB‐10 and PSB13078g2 from the filamentous cyanobacterium CCP2, retained the WXDXXL motif (Figure 1c, d).

### 2.2 Spectral properties of novel XRG CBCR GAF domains

The novel XRG CBCR GAF domains were expressed in PCB‐producing *Escherichia coli* and purified using nickel affinity chromatography. SDS‐PAGE CBB staining revealed multiple protein bands, with the band around 20 kDa aligning closely with the theoretical molecular sizes of the GAF domains (Figure S2). Notably, only these bands exhibited fluorescence in the presence of zinc ions, indicating that they represent the chromophorylated GAF domains (Figure S2).

**FIGURE 2:**
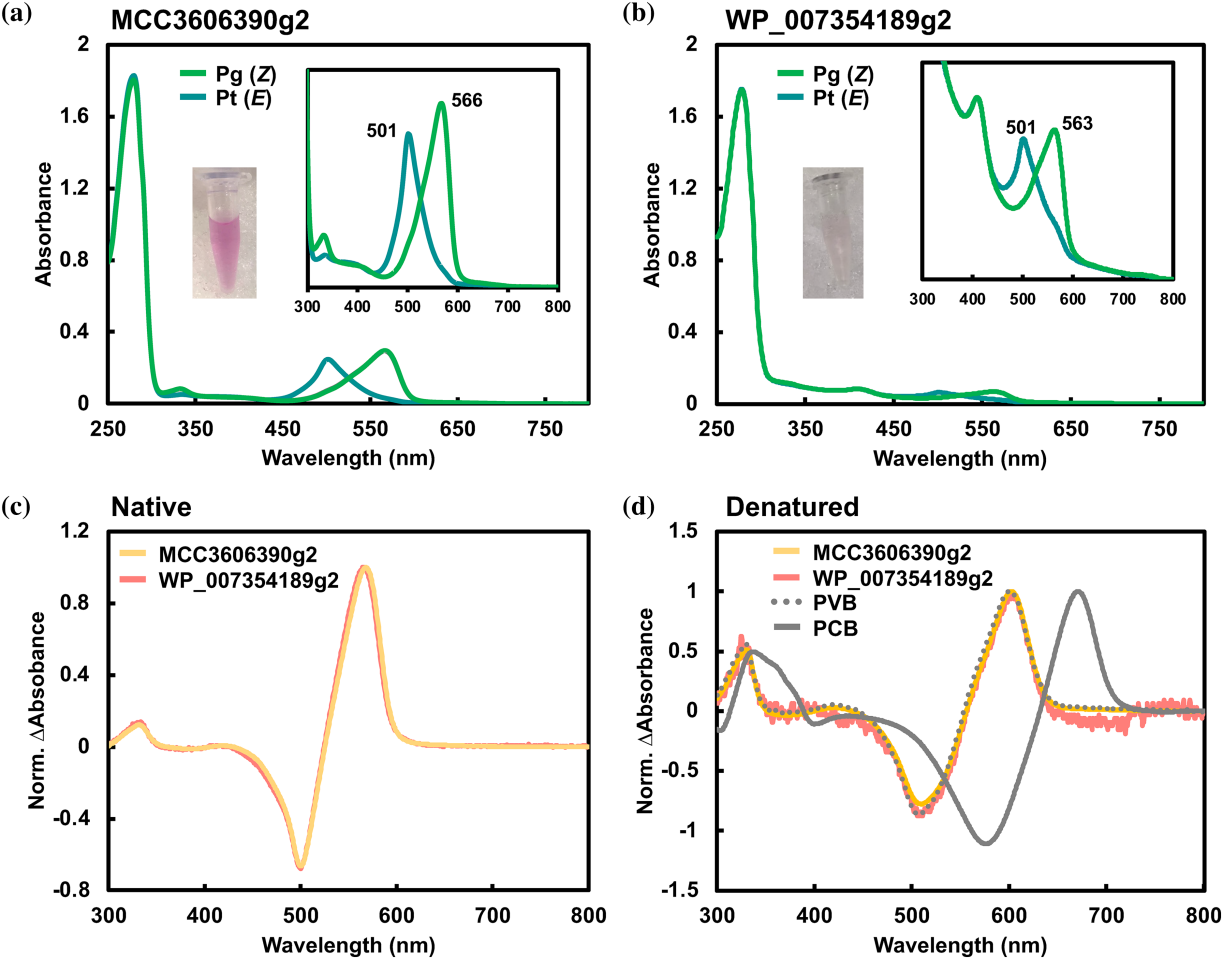
Absorption spectra of the novel PVB‐binding XRG CBCR GAF domains. (a) Absorption spectra of native MCC3606390g2. (b) Absorption spectra of native WP_007354189g2. (c) Normalized difference spectra of native MCC3606390g2 (orange) and WP_007354189g2 (salmon pink). (d) Normalized difference spectra of denatured MCC36063390g2 (orange) and WP_007354189g2 (salmon pink) with reference samples of PCB‐binding (AM1_C0023g2, gray‐dash) (Fushimi et al., 2016) and PVB‐binding ones (AM1_6305g1, gray‐solid) (Hasegawa et al., 2018).

MCC3606390g2 exhibited reversible photoconversion between a Pg state peaking at 566 nm and a Pt state peaking at 501 nm (Figure 2a). Similarly, WP_007354189g2 displayed reversible photoconversion between a Pg state peaking at 563 nm and a Pt state peaking at 501 nm (Figure 2b). These Pg‐minus‐Pt difference spectra were nearly identical (Figure 2c). Acid denaturation analysis indicated that the Pg states were dark‐adapted states binding the *Z*‐configured PVB, while the Pt states were photoproduct states binding the *E*‐configured PVB in both cases (Figures 2d and S3A–D). Notably, despite belonging to the XRG lineage and lacking the DXCF second Cys residue (Figure 1b, d), both of these CBCR GAF domains displayed PCB‐to‐PVB isomerization activity. A relatively small spectral shift 62–65 nm was observed for green/teal photoconversion, suggesting that these molecules do not exhibit reversible Cys ligation such as blue/green CBCR GAF domains (Ishizuka et al., 2011). To confirm this, iodoacetamide (IAM) was added to both states of MCC3606390g2 (Figure S4). The addition of IAM did not affect either the Pg‐to‐Pt or Pt‐to‐Pg photoconversion, leading to the conclusion that the green/teal photocycle occurs without reversible Cys adduct formation.

**FIGURE 3:**
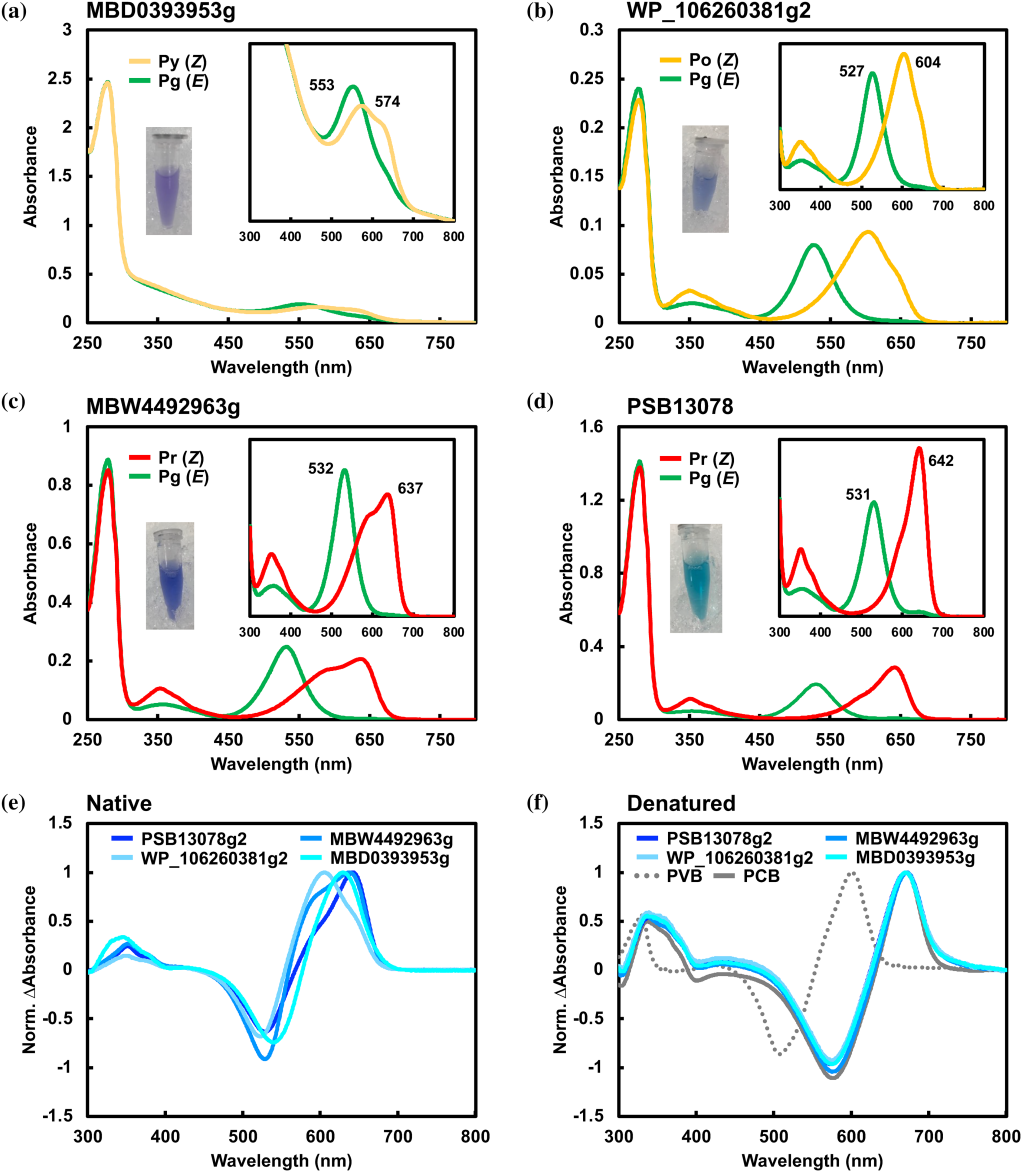
Absorption spectra of the PCB‐binding XRG CBCR GAF domain. (a) Absorption spectra of native MBD0393953g. (b) Absorption spectra of native WP_106260381g2. (c) Absorption spectra of native MBW4492963g. (d) Absorption spectra of native PSB13078g2. (e) Normalized difference spectra of native PSB13078g2 (blueberry), MBW4492963g (aqua blue), WP_106260381g2 (sky blue), and MBD0393953g (turquoise blue). (f) Normalized difference spectra of denatured PSB13078g2 (Blueberry), MBW4492963g (aqua blue), WP_106260381g2 (sky blue), and MBD0393953g (turquoise blue) with reference samples of PCB‐binding (AM1_C0023g2, gray‐dash) (Fushimi et al., 2016) and PVB‐binding ones (AM1_6305g1, gray‐solid) (Hasegawa et al., 2018).

MBD0393953g and WP_106260381g2 both featuring the same CXDXXL motif, displayed reversible photoconversion between an orange‐ (Po) or a yellow‐absorbing (Py) dark‐adapted state (λ_max_ = 574 and 604 nm, respectively) binding the *Z*‐configured PCB, and a Pg photoproduct state (λ_max_ = 553 and 527 nm, respectively) binding the *E*‐configured PCB (Figures 3a, b, e, f and S3E–H). This contrasts with the results of MCC3606390g2 and WP_007354189g2 (Figures 2, and S3A–D). Notably, these CBCR GAF domains did not demonstrate PCB‐to‐PVB isomerization activity, suggesting that the Cys residue of the CXDXXL motif might not be sufficiently functional in the PCB‐to‐PVB isomerization process (Figure 3f). It is possible that MCC3606g2 and WP_007354189g2 possess unique amino acid residues crucial for PCB‐to‐PVB isomerization.

MBW4492963g and PSB13078g2, featuring the canonical WXDXXL motif, demonstrated reversible photoconversion between the Pr dark‐adapted states (λ_max_ = 637 and 642 nm, respectively) binding the *Z*‐configured PCB and the Pg photoproduct states (λ_max_ = 532 and 531 nm, respectively) binding the *E*‐configured PCB, which were typical for XRG molecules (Figure 3c–f and Figure S3I–L).

### 2.3 Identification of amino acid residues crucial for PCB‐to‐PVB isomerization

**FIGURE 4:**
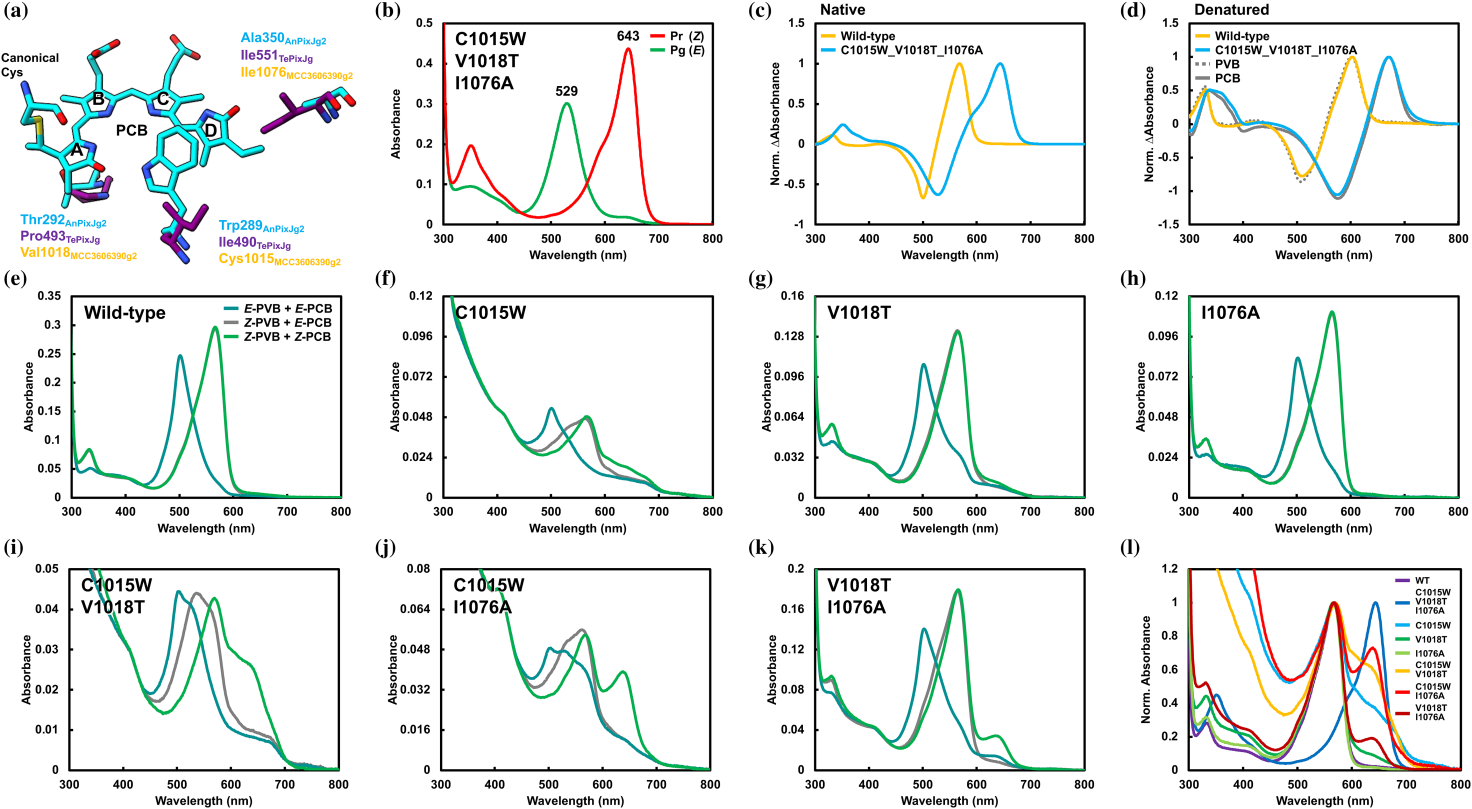
Site‐directed mutagenesis on MCC3606390g2. (a) Structure comparison between MCC3606390g2 and the typical CBCR GAF domains. Residues crucial for PCB‐to‐PVB isomerization activity were highlighted on AnPixJg2 (blue, PDB ID: 3W2Z) and TePixJg (purple, PDB ID: 4GLQ) structures. (b) Absorption spectra of the triple mutant, MCC3606390g2_C1015W_V1018T_I1076A. (c) Normalized difference spectra of native MCC3606390g2 wild‐type (orange) and MCC3606390g2_C1015W_V1018T_I1076A (blue). (d) Normalized difference spectra of denatured MCC3606390g2 wild‐type (orange) and MCC3606390g2_C1015W_V1018T_I1076A (blue) with reference samples of PVB‐binding (AM1_6305g1, gray‐solid) (Hasegawa et al., 2018) and PCB‐binding ones (AM1_C0023g2, gray‐dash) (Fushimi et al., 2016). (e)–(k) Absorption spectra of native MCC3606390g2 wild‐type (e), C1015W (f), V1018T (g), I1076A (h), C1015W_V1018T (i), C1015W_I1076A (j), and V1018T_I1076A (k). Green lines represent absorption spectra of mixtures of the *Z*‐configured PVB‐ and PCB‐binding components. Gray lines represent absorption spectra of mixtures of the *Z*‐configured PVB‐ and *E*‐configured PCB‐binding components. Teal lines represent absorption spectra of mixtures of *E*‐configured PVB‐ and PCB‐binding components. (l) Normalized absorption spectra of mixtures of Z‐configured PVB‐ and PCB‐binding components of the mutants.

To identify amino acid residues crucial for PCB‐to‐PVB isomerization, we conducted a multiple sequence alignment of six XRG CBCR GAF domains found in this study with typical XRG CBCR GAF domains (Figure 1d). Based on this alignment, we focused on three amino acid residues, Cys, Val, and Ile residues (Cys1015/Val1018/Ile1076 of the MCC3606390g2) (Figure 1d and Figure 4a). The Cys residue within the CXDXXL motif is conserved among the two PVB‐binding molecules (MCC3606390g2 and WP_007354189g2) and the two PCB‐binding molecules (MBD0393953g and WP_106260381g2), while the other two PCB‐binding molecules (MBW4492963g and PSB13078g2) and typical PCB‐binding molecules have Trp residues at this position (Figure 1d). In contrast, the Val and Ile residues are exclusively conserved among the two PVB‐binding molecules, whereas the other four PCB‐binding molecules have Thr and Ala residues at these positions (Figure 1d).

The triple mutant MCC3606390g2_C1015W_V1018T_I1076A exhibited photoconversion between a Pr dark‐adapted state peaking at 643 nm and a Pg photoproduct state peaking at 529 nm, similar to typical XRG CBCR GAF domains (Figure 4b). No detectable green/teal photoconversion components were observed (Figure 4c). Acid denaturation analysis revealed that the chromophore bound to the triple mutant was not PVB but PCB (Figure 4d). In conclusion, this mutant molecule completely lost its PCB‐to‐PVB isomerization activity. To investigate the role of each amino acid residue, single and double mutants were constructed and subjected to spectroscopic analysis (Figures 4e–k, S2, S5, and S6). These mutants displayed heterogeneous binding behaviors to both PVB and PCB, as indicated by the absorption spectra of the native and denatured samples (Figures 4e–k, S5, and S6). To accurately evaluate these heterogeneous samples, we established a light illumination protocol to photoconvert one of the PVB‐ and PCB‐binding components specifically (see the “Materials and Methods” section in detail). This protocol generated three preparations: (A) PVB‐ and PCB‐binding *Z*‐isomers, (B) PVB‐binding *Z*‐isomer and PCB‐binding *E*‐isomer, and (C) PVB‐ and PCB‐binding *E*‐isomers (Figure S7). The absorption spectra of preparations A‐to‐C were summarized in Figure 4. The *Z*‐minus‐*E* difference absorption spectra of the PVB‐binding component (A‐minus‐B) and the PCB‐binding component (B‐minus‐C) were summarized in Figure S6. We first applied this protocol to the wild‐type molecule and detected a negligible red/green photoconvertible component (Figures 4e and S6A, H). The single mutant I1076A exhibited green/teal reversible photoconversion with a faint red/green reversible component similar to the wild‐type molecule (Figures 4h and S6D, K), indicating that I1076A binds PVB like the wild‐type molecule, displaying almost full PCB‐to‐PVB isomerization activity. In contrast, the C1015W and V1018T mutant molecules contained larger amounts of PCB‐binding components than the I1076A mutant molecule (Figures 4f, g and S6B, C, I, J). The double mutants C1015W_V1018T, C1015W_I1076A, and V1018T_I1076A contained larger amounts of PCB‐binding components than the corresponding single mutants (Figures 4i–k and S6E–G, L–N). All double mutants accumulated significant levels of PVB‐binding components, suggesting that all three amino acid residues, Cys1015, Val1018, and Ile1076 of MCC3606390g2, are crucial for the full PCB‐to‐PVB isomerization ability.

To quantitatively assess the PCB‐to‐PVB isomerization activity, we normalized the spectra of preparation A, where both the PVB‐ and PCB‐binding components were present as dark‐adapted states (Figure S7), by the peak absorbance of the Pg dark‐adapted states of the PVB‐binding components (Figure 4l). However, some mutations affect the absorption shape of the PCB‐binding component, preventing quantitative evaluation. Instead, we calculated the ratio of PVB to PCB in the variant molecules from the spectra of acid‐denatured preparations as previously reported (Figure S5 and Table 1) (Ma et al., 2012). The amount of the PCB‐binding component in the wild‐type was estimated as 1.8%, indicating almost full PCB‐to‐PVB isomerization activity. Among the single mutants, the C1015W mutant contained the largest amount of the PCB‐binding component (14.7%) compared to the V1018T and I1076A mutants (6.9% and 0.6%, respectively). Consistently, the double mutants containing the C1015W mutation (C1015W_V1018T, C1015W_I1076A) contained a greater number of PCB‐binding components (45.5% and 33.9%, respectively) than the V1018T_I1076A double mutant (7.4%). C1015W mutation had the most significant inhibitory effect on the PCB‐to‐PVB isomerization activity. Furthermore, for both the single and double mutants, V1018T mutation (6.9% and 45.5% for the V1018T single and C1015W_V1018T double mutants, respectively) showed more significant inhibitory effect on the PCB‐to‐PVB isomerization activity than I1076A mutation (0.6% and 33.9% for the I1076A single and C1015W_ I1076A double mutants, respectively). Not simple additive effects but synergetic effects were observed for the double and triple mutants except for the V1018T_I1076A double mutant (7.4%). In conclusion, Cys1015 appears to be the most crucial, while Val1018 and Ile1076 seem to play supportive roles with higher contribution of Val1018.

**TABLE 1:** The ratio of PVB and PCB incorporated into MCC3606390g2 variant molecules.

MCC3606390g2	ABS at 592 nm	ABS at 662 nm	PVB (%)	PCB (%)
Wild‐type	0.1675	0.0063	98.2	1.8
C1015W	0.0039	0.0012	85.3	14.7
V1018T	0.0723	0.0105	93.1	6.9
I1076A	0.0519	0.0007	99.4	0.6
C1015W_V1018T	0.0227	0.0217	54.5	45.5
C1015W_I1076A	0.0059	0.0042	66.1	33.9
V1018T_I1076A	0.0245	0.0038	92.6	7.4

*Note*: These ratios were estimated from absorbances at 592 nm (*Z*‐PVB) and 662 nm (*Z*‐PCB) of the denatured absorption spectra (Figure S5) as previously reported (Ma et al., 2012).

### 2.4 Introduction of key amino acid residues into other XRG CBCR GAF domains

The three amino acid residues responsible for PCB‐to‐PVB isomerization ability in MCC3606390g2 were introduced into the other XRG CBCR GAF domains, WP_106260381g2 and AnPixJg2, to determine if these three residues alone are sufficient for the PCB‐to‐PVB isomerization (Figure 1d). However, the purified WP_10620381g2_T566V_A624I and AnPixJg2_W289C_T292V_A350I mutant molecules did not exhibit zinc‐induced fluorescence in the SDS‐PAGE gel or specific absorbance in the visible light region, except for a faint heme‐like absorption, likely due to the contaminant protein from *E. coli* (Figures S2 and S8). In conclusion, these mutants failed to bind any chromophore, preventing the evaluation of the effect of these amino acid residues on PCB‐to‐PVB isomerization activity.

## 3 DISCUSSION

Despite belonging to the XRG lineage, MCC3606390g2 and WP_007354189g2 bound PVB instead of PCB, suggesting that these two CBCR GAF domains acquired PCB‐to‐PVB isomerization ability through alterations in their amino acid residues during the molecular evolution process (Figures 1 and 2). Site‐directed mutagenesis revealed that replacing three amino acid residues (C1015W, V1018T, and I1076A) with those from MBW4492963g and PSB13078g2 led to a complete loss of PCB‐to‐PVB isomerization ability (Figure 4b–d).

**FIGURE 5:**
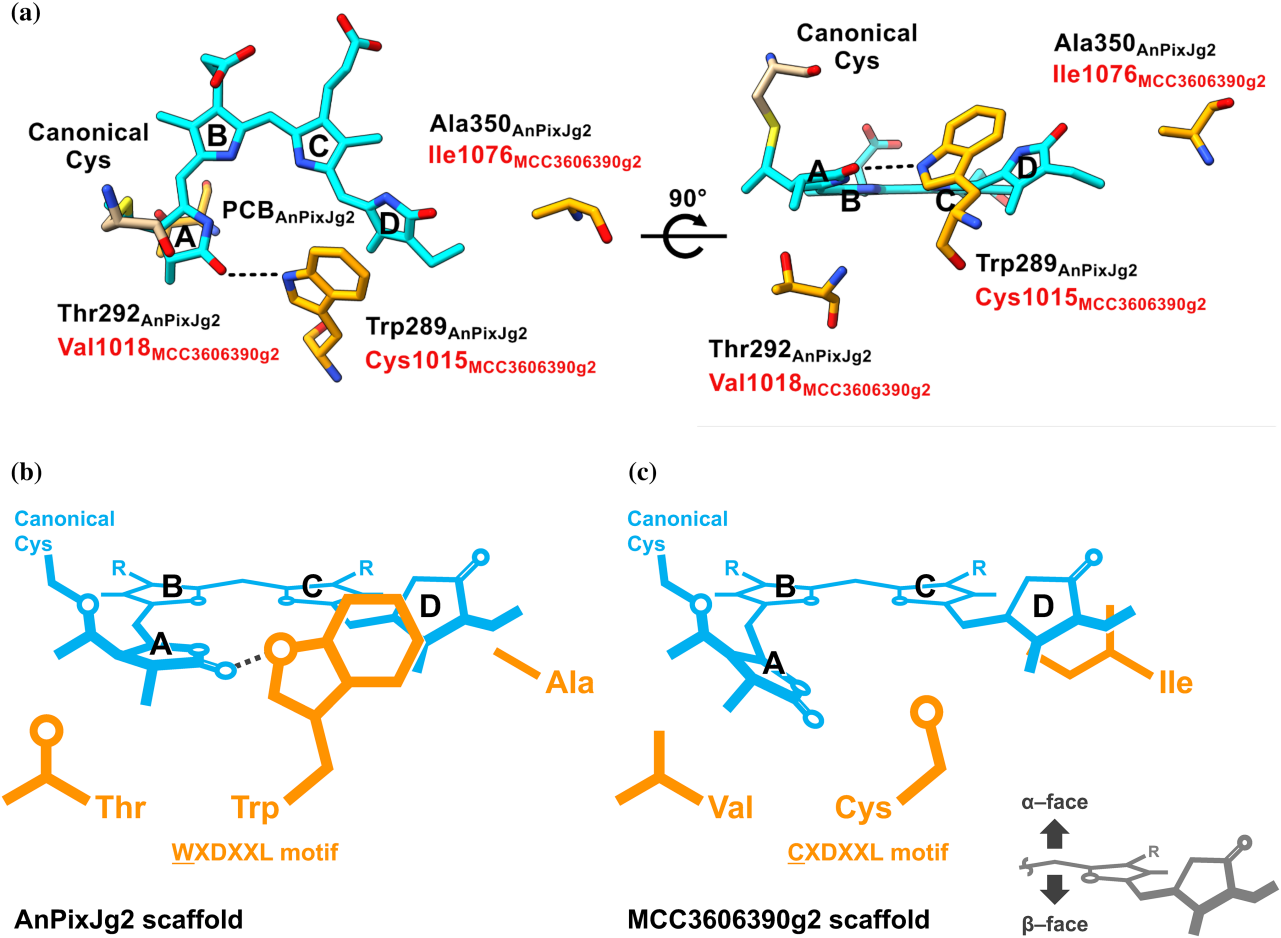
Amino acid residues crucial for the PCB‐to‐PVB isomerization in MCC3606390g2. (a) The three residue positions highlighted on the structure of the Pr dark‐adapted state of AnPixJg2 (PDB ID: 3W2Z). (b) Conformation of PCB incorporated into AnPixJg2 scaffold having WXDXXL motif. (c) Predicted conformations of PCB or PVB incorporated into MCC3606390g2 scaffold having CXDXXL motif. The dashed line indicates the hydrogen bond between the side chain of Trp of the WXDXXL motif and the hydroxyl group of the A‐ring of PCB.

To explore the contribution of these three amino acid residues to PCB‐to‐PVB isomerization activity, we examined the corresponding residues of the Pr dark‐adapted state structure of AnPixJg2 (Figure 5a) (Narikawa et al., 2013). Site‐directed mutagenesis revealed that replacing Cys1015 with Trp had the most significant inhibitory effect on PCB‐to‐PVB isomerization (Figure 4). In the Pr dark‐adapted state of AnPixJg2, the corresponding Trp residue forms a π–π stacking with the D‐ring and a hydrogen bond with the A‐ring nitrogen, crucial for maintaining a stable chromophore conformation to absorb red light (Figures 5a and S1C). However, the Cys residue would lack both interactions. Consequently, the A‐ring, not stabilized by the hydrogen bond, may be twisted relative to the B–C plane of the rings (Figure 5b, c). Homologous molecules, MBD0393953g and WP_106260381g2 molecules possessing the same CXDXXL motif did not exhibit PCB‐to‐PVB isomerization ability, likely due to the influence of the other two residues (Figures 1 and 3f). The dark‐adapted states of these molecules absorbed in the yellow‐to‐orange region, shorter than the red absorption of typical red/green reversible molecules like AnPixJg2 (Figure 3a, b). This blue‐shifted feature would result from the A‐ring twist due to the absence of the Trp residue.

MCC3606390g2 features a Val1018 residue near the A‐ring (Figure 5a), which, in addition to Cys1015, plays a crucial role in PCB‐to‐PVB isomerization (Figure 4). Val1018 likely contributes to stabilizing the twisted A‐ring through hydrophobic interaction to a greater extent. In vitro reconstitution analysis with the PCB chromophore has shown that the typical DXCF CBCR GAF domain, TePixJg, binds PCB immediately after reconstitution and undergoes blue/green reversible photoconversion (Ishizuka et al., 2011). TePixJg demonstrates both PCB‐to‐PVB isomerization activity and reversible Cys ligation, where the second Cys residue binds to the C10 of the chromophore in the dark‐adapted state of Pb to absorb blue light. In this context, the blue light absorption in the dark‐adapted state incorporating the PCB chromophore immediately after reconstitution is likely due to Cys adduct formation between C10 of PCB and the second Cys residue. On the other hand, green light absorption in the photoproduct state, even after incorporating the PCB chromophore immediately after reconstitution, may be attributed to the high distortion of the A‐ring. This highly distorted A‐ring is believed to be a trigger for PCB‐to‐PVB isomerization (Fushimi & Narikawa, 2021b). A similar A‐ring twist, facilitated by Cys1015 and Val1018, is presumed to also trigger PCB‐to‐PVB isomerization in the MCC3606390g2 molecule.

Ile1076, corresponding to the Ala residue in AnPixJg2, is situated at the β‐face of the D‐ring, opposite to the Cys/Trp residue (Figure 5a). The bulky and hydrophobic side chain of Ile1076 contributes to stabilizing the D‐ring conformation through hydrophobic interaction, complementing the role of the Trp residue on the opposite side. In typical DXCF CBCR GAF domains, PCB‐to‐PVB isomerization occurs under dark conditions, with the second Cys residue ligating to C10 of the chromophore (Ishizuka et al., 2011). In this context, the high distortion of the C4 = C5 double bond is established by the dual fixation of the chromophore at the A‐ring and C10 position via the first and second Cys residues, respectively. This highly unstable and rigid conformation would trigger reduction at the C4 = C5 double bond and concomitant oxidation of the C2–C3 single bond of the A‐ring (Fushimi & Narikawa, 2021b; Rockwell, Martin, Gulevich, & Lagarias, 2012). As the MCC3606390g2 molecule did not exhibit reversible Cys adduct formation with the C10 of the chromophore, an alternative method must establish the high distortion of the C4 = C5 double bond. We recently reported that the DXCF CBCR molecule AM1_6305g1 did not require the second Cys residue for PCB‐to‐PVB isomerization (Fushimi & Narikawa, 2021b). Replacement of the second Cys with Ser did not affect the isomerization activity. In this molecule, amino acid residues near the D‐ring would complement the role of the second Cys by firmly fixing the D‐ring but not the C10 of the chromophore. Ile1076 in MCC3606390g2 would similarly contribute to the high distortion of the C4 = C5 double bond by fixing the D‐ring.

Although highly twisted A‐ring has been observed for the Pg photoproduct state of the red/green CBCR GAF domains (Lim et al., 2018; Xu et al., 2020), these domains do not show PCB‐to‐PVB isomerization activity at all (Chen et al., 2012; Narikawa et al., 2008; Rockwell, Martin, & Lagarias, 2012c). Because the dark‐adapted state but not the photoproduct state should be first generated when the holoprotein has been folded, PCB‐to‐PVB isomerization within the CBCR GAF domains would occur in the dark‐adapted states. In this context, highly twisted A‐ring in the Pg photoproduct state would not result in PCB‐to‐PVB isomerization.

Among the different MCC3606390g2 mutant molecules, the C1015W and C1015W_V1018T mutants with Trp and Ile residues at the Cys1015 and Ile1076 positions, respectively, exhibited a broad orange‐to‐red absorption in the dark‐adapted states for the PCB‐binding components, whereas the other mutants displayed sharp red absorption (Figure 4e–k). Bulky residues (Trp and Ile) on both the α‐ and β‐faces of the D‐ring could create a confined environment around the D‐ring, potentially resulting in broad absorption.

The V1018T_I1076A double mutant of MCC3606390g2 shares the same combination of amino acid residues as the homologous proteins MBD0393953g and WP106260381g2, which are crucial for PCB‐to‐PVB isomerization (Figure 1d). Notably, the V1018T_I1076A double mutant retained partial PCB‐to‐PVB isomerization activity, whereas the wild‐type molecules MBD0393953g and WP10620381g2 did not exhibit detectable isomerization activity. This suggests that MCC3606390g2 may possess additional unidentified amino acid residue(s) crucial for PCB‐to‐PVB isomerization activity (Figures 3a, b, f, 4k, and S6G, N). Further comparisons based on sequence and structure would reveal additional residues in the future.

A previous study identified Lyn8106_0097 and Lyn.aest_0230, referred to as RB2 with CXDXXL motifs, as equivalent to MCC3606390g2 and WP_007354189g2. These RB2 molecules bind PCB and undergo red/blue photocycles, indicating the formation of a covalent bond to the C10 of the chromophore during photoconversion through the Cys residue within the motif (Blain‐Hartung et al., 2021). While the Cys residue at the same position reversibly binds to the C10 of the chromophore in the RB2 molecules, it contributes to PCB‐to‐PVB isomerization in MCC3606390g2 and WP_007354189g2. RB2 molecules belong to the NpR3784 lineage rather than the XRG lineage (Figure 1a). This NpR3784 lineage, like the XRG lineage, lacks the second Cys residue within the DXCF motif, and its typical molecules exhibit a red/green photocycle similar to XRG CBCR molecules. The typical molecules of the NpR3784 lineage do not have a Trp residue at the Cys position within the CXDXXL motif. Instead, the Val residue is highly conserved among the NpR3784 lineage, and the interaction network via this residue is likely different from that via the Trp residue in the XRG lineage, potentially resulting in different functionality of the Cys residue at the same position.

The PCB‐binding MCC3606390g2 triple mutant molecule undergoes red/green reversible photoconversion with a spectral shift of over 100 nm, while the PVB‐binding MCC3606390g2 wild‐type molecule undergoes green/teal reversible photoconversion with only a 65 nm spectral shift (Figures 2a and 4b). This difference likely arises from differences in the chromophore structure. The PCB chromophore has four tetrapyrrole rings that are fully conjugated, whereas the A‐ring of the PVB chromophore is deconjugated from the other rings (Figure S1A). In typical PCB‐binding XRG molecules, both rings A and D exhibit high levels of twisting relative to the rings B–C plane in the Pg state compared to the Pr state (Lim et al., 2018 ; Xu et al., 2020), resulting in a significant blue shift. However, PVB incorporation cancels the effect of A‐ring distortion on absorption, leading to a smaller spectral shift.

## 4 MATERIALS AND METHODS

### 4.1 Bioinformatics

We searched for novel CBCR GAF domain sequences using blast (protein BLAST) and PSI‐BLAST (position‐specific iterated BLAST) in the NCBI database. The acquired sequences underwent multiple sequence alignment using MEGA11 (Tokyo Metropolitan University, Tokyo, Japan) (Tamura et al., 2021) and MAFFT (Osaka University, Osaka, Japan) (Katoh et al., 2019). For constructing a phylogenetic tree, we utilized MAFFT v7.505 [‐‐genafpair ‐‐maxiterate 1000] for aligning sequences and IQ‐TREE v2.2.2.7 [−bb 1000, −alrt 1000] for maximum‐likelihood phylogenetic analysis (Nguyen et al., 2015). To select the substitution model, we employed IQ‐TREE's built‐in ModelFinder and opted for Q.pfam+R9, which had the lowest Bayesian information criterion score among 616 models. Statistical robustness was evaluated using the ultrafast bootstrap (Hoang et al., 2018) and the SH‐aLRT test (Guindon et al., 2010). Molecular graphics of AnPixJg2 in the Pr state (PDB ID: 3W2Z) and TePixJg in the Pb state (PDB ID: 4GLQ) was generated using the UCSF ChimeraX software (University of California, San Francisco, USA) (Goddard et al., 2018).

### 4.2 Plasmid construction

Plasmids were created for protein expression in *E. coli* by inserting gene fragments of MCC3606390g2 (MCC3606390, amino acid positions: 962–1123), WP_007354189g2 (WP_007354189, amino acid positions: 1100–1171), MBD0393953g (MBD0393953 partial, amino acid positions: 28–199), WP_106260381g2 (WP_106260381, amino acid positions: 500–671), MBW4492963g (MBW4492963, amino acid positions: 704–875), and PSB13078g2 (PSB13078, amino acid positions: 996–1167). These gene fragments, fused with the His‐tag sequence on their N‐terminal, were inserted into the pET151‐D‐TOPO vector (Invitrogen, ThermoFisherScientific, Massachusetts, USA). The gene fragments were artificially synthesized (ThermoFisherScientific, Massachusetts, USA), and codons optimized for expression in *E. coli*. KOD One PCR Master Mix (TOYOBO, Osaka, Japan) was employed to introduce mutations into MCC3606390 using appropriate primers (Table S1).

### 4.3 Protein expression and purification

The proteins were expressed in *E. coli* C41 pKT271_C0185, which was incubated at 37°C until the OD_600_ reached a range of 0.4–0.8. Isopropyl‐β‐D‐thiogalactopyranoside was then added to induce expression at 18°C. The cells were collected by centrifugation at 5000× *g* for 20 min and frozen at −80°C for 30 min or longer. Afterward, the cells were suspended in a lysis buffer (20 mM HEPES‐NaOH pH 7.5, 0.1 M NaCl, and 10% (w/v) glycerol) and disrupted using an Emulsiflex C5 high‐pressure homogenizer at 12,000 psi (Avestin, Inc., Ottawa, Canada, ON, Canada). The mixtures were centrifuged at 17,000× *g* for 60 min to separate the pellets and supernatants. The collected supernatants were filtered through a 0.8‐μm cellulose acetate membrane and loaded onto a nickel‐affinity His‐trap column (GE Healthcare, Piscataway, NJ, USA) using ÄKTA pure (GE Healthcare, Piscataway, NJ, USA). His‐tagged proteins were purified using a lysis buffer containing 100–400 mM imidazole with a linear gradient system (1 mL/min, 15 min) after the column was washed using a lysis buffer containing 100 mM imidazole. EDTA (final concentration, 1 mM) was added to the purified protein, which was incubated on ice for 1 h and then dialyzed against the lysis buffer to remove imidazole and EDTA.

### 4.4 Spectral analysis

UV‐2600 spectrophotometer (SHIMADZU, Kyoto, Japan) was used to record the ultraviolet and visible absorption spectra of the native proteins. An Opto‐Spectrum Generator (Hamamatsu Photonics, Inc., Hamamatsu, Japan) was utilized to produce monochromic light for photoconversion. Denaturation of proteins was achieved using 1 M HCl/8 M urea and the absorption spectra of the denatured proteins were measured at room temperature. The *Z*‐configured PCB‐ and PVB‐binding components of the MCC3606390g2 mutants were generated by irradiation with 500 nm light for 1 min (Figure S7A). A mixture of the *E*‐configured PCB‐binding component and the *Z*‐configured PVB‐binding component of the MCC3606390g2 mutants was obtained by successive irradiation with 500 nm light for 1 min and 650 nm light for 1 min (Figure S7B). Additionally, a mixture of *E*‐configured PCB‐ and PVB‐binding components of the MCC3606390g2 mutants was generated by successive irradiation with 590‐nm light for 1 min and 650‐nm light for 1 min (Figure S7C). The ratio of PCB and PVB bound to the MCC3606390g2 mutants were estimated from absorbances at 592 nm derived from *Z*‐PVB and 662 nm derived from *Z*‐PCB of these denatured absorption spectra (Figure S5), based on the calculation method presented in the previous study (Ma et al., 2012).

### 4.5 Electrophoresis

The purified proteins were diluted in a buffer (60 mM dithiothreitol (DTT), 2% (w/v) sodium dodecyl sulfate (SDS), and 60 mM Tris–HCl pH 8.0) for SDS‐PAGE. Following denaturation by heat shock at 95°C for 3 min, the samples were electrophoresed at room temperature using a 12% (w/v) acrylamide gel. Subsequently, the electrophoresed gels were immersed in 20 mM zinc acetate at room temperature for 30 min to visualize the fluorescence of the purified proteins. Blue light (λ_max_ = 470 nm) and green light (λ_max_ = 527 nm) were used for illumination, and a WSE‐5500 VariRays (ATTO, Tokyo, Japan) equipped with a short path filter (passing through <562 nm) and a long path filter (passing through >600 nm) was utilized to capture the fluorescence bands. The fluorescence bands were then imaged using a WSE109 6100 LuminoGraph (ATTO, Tokyo, Japan). Subsequently, the gels were stained with Coomassie brilliant blue R‐250 (CBB) after observation.

## AUTHOR CONTRIBUTIONS

**Hiroki Hoshino:** Conceptualization; methodology; investigation; writing – original draft; data curation; writing – review and editing; validation. **Keita Miyake:** Methodology; data curation; investigation; writing – review and editing; validation. **Keiji Fushimi:** Methodology; writing – review and editing; writing – original draft; investigation; validation; data curation. **Rei Narikawa:** Conceptualization; supervision; funding acquisition; writing – original draft; writing – review and editing.

## CONFLICT OF INTEREST STATEMENT

The authors have no conflicts of interest to disclose regarding this manuscript.

## Supporting information


**Data S1** Supporting Information

## Data Availability

No data from this study have been deposited in external repositories.
