# Supplementary material for: Red/green cyanobacteriochromes acquire isomerization from phycocyanobilin to phycoviolobilin
Source: Protein Sci. 2024 Jul 29;33(8):e5132. doi: 10.1002/pro.5132 (PMC11284453; doi:10.1002/pro.5132)
Supplement: Supplementary file 1 — Data S1 Supporting Information [file PRO-33-e5132-s001.docx]

Fig. S1. Photoconversion mechanisms in typical cyanobacteriochromes (CBCRs). (A) Chemical structures of phycoviolobilin (PVB, upper) and phycocyanobilin (PCB, lower). These chromophores are incorporated into tetrapyrrole-binding photoreceptors and exhibit reversible isomerization between the 15*Z* and 15*E* forms by sensing light. (B) Reversible photoconversion mechanism in typical blue/green CBCRs. The second Cys residue within the Asp-Xaa-Cys-Phe (DXCF) motif forms a covalent bond with the C10 of the *Z*-configured PVB but is detached from the C10 of the *E*-configured PVB. (C) Reversible photoconversion mechanism in typical red/green CBCRs. The Trp residue within the Trp-Xaa-Asp-Xaa-Xaa-Leu (WXDXXL) motif forms a hydrogen bond and a π-π stacking with the A- and D-rings of the *Z*-configured PCB, respectively. In contrast, these interactions are resolved for the *E*-configured PCB, resulting in highly twisted A- and D-rings. The π-conjugated systems of PVB and PCB were shown in violet and blue, respectively.

Fig. S2. SDS-PAGE of purified CBCR GAF domains. The XRG CBCR GAF domains were detected by CBB staining (upper) and Zinc-induced fluorescence (lower).

Fig. S3. Absorption spectra of denatured XRG CBCR GAF domains analyzed in this study. Each sample was denatured using 1 M HCl/8M urea. After denaturation, white light was irradiated onto these samples. Colored lines represent spectra just after denaturation, while black lines represent spectra after light illumination. (A-B) Absorption spectra of native MCC3606390g2 in the Pg state (A) and Pt state (B). (C-D) Absorption spectra of denatured WP_007354189g2 in the Pg state (C) and Pt state (D). (E-F) Absorption spectra of denatured MBD0393953g in the Py state (E) and Pg state (F). (G-H) Absorption spectra of denatured WP_106260381g2 in the Po state (G) and Pg state (H). (I-J) Absorption spectra of denatured MBW4492963g in the Pr state (I) and Pg state (J). (K-L) Absorption spectra of denatured PSB13078g2 in the Pr state (K) and Pg state (L).

Fig. S4. Iodoacetamide (IAM) treatment of MCC3606390g2. IAM was added to MCC3606390g2 in the Pg dark-adapted state (A) and the Pt photoproduct state (B). After adding IAM, the samples were kept in the dark for 5 min, followed by irradiation with green or teal light.

Fig. S5. Absorption spectra of denatured MCC3606390g2 variant molecules. Each sample was denatured using 1 M HCl/8M urea after light irradiation according to the protocol shown in Fig. S7. MCC3606390g2 wild-type (A), C1015W (B), V1018T (C), I1076A (D), C1015W_V1018T (E), C1015W_I1076A (F), and V1018T_I1076A (G). Green lines represent absorption spectra of mixtures of the *Z*-configured PVB- and PCB-binding components. Gray lines represent absorption spectra of mixtures of the *Z*-configured PVB- and *E*-configured PCB-binding components. Teal lines represent absorption spectra of mixtures of *E*-configured PVB- and PCB-binding components.

Fig. S6. Normalized difference spectra of native and denatured MCC3606390g2 variant molecules. Normalized difference spectra of native wild-type (A), C1015W (B), V1018T (C), I1076A (D), C1015W_V1018T (E), C1015W_I1076A (F), and V1018T_I1076A (G). The spectra of MCC3606390g2 mutants corresponding to the PVB- and PCB-binding components are colored purple and blue, respectively. These difference spectra were calculated from the spectra shown in Fig. 4E-K. Normalized difference spectra of denatured wild type (H), C1015W (I), V1018T (J), I1076A (K), C1015W_V1018T (L), C1015W_I1076A (M), and V1018T_I1076A (N). The spectra corresponding to the PVB- and PCB-binding components of the MCC3606390g2 mutants are colored blue and purple, respectively, whereas the PVB- and PCB-binding reference samples (AM1_6305g1 and AM1_C0023g2)^23,31^ are indicated by gray solid and dash lines, respectively. These difference spectra were calculated from the spectra shown in Fig. S5.

Fig. S7. Light illumination protocol for targeted photoconversion of PVB- and PCB-binding components. This protocol generated three preparations: (A) PVB-binding *Z*-isomer and PCB-binding *Z*-isomer, (B) PVB-binding *Z*-isomer and PCB-binding *E*-isomer, and (C) PVB-binding *E*-isomer and PCB-binding *E*-isomer.

Fig. S8. Introduction of key amino acid residues for chromophore isomerization from PCB to PVB in MCC3606390g2 to PCB-binding XRG CBCR GAF domains. Absorption spectra of (A) WP_106260381g2_T566V_A624I and (B) AnPixJg2_W289C_T292V_A350I.

Table S1. Primer sets for site-directed mutagenesis.

| **CBCR GAF domain** | **Mutation** | **Sequence** | |
| --- | --- | --- | --- |
| **MCC3606390g2** | **C1015W** | **Fw** | 5'-AAAGCCtggGAAGATGTGTATCTGCAAGAAACCAAAGG-3' |
|  |  | **Rv** | 5'-ATCTTCccaGGCTTTCTGAATATTGCATTCAACCAG-3' |
|  | **V1018T** | **Fw** | 5'-GAAGATaccTATCTGCAAGAAACCAAAGGTGGTCG-3' |
|  |  | **Rv** | 5'-CAGATAggtATCTTCGCAGGCTTTCTGAATATTGC-3' |
|  | **I1076A** | **Fw** | 5'-CTGCTGgcgATCTATCAGAATACCGCACCGCG-3' |
|  |  | **Rv** | 5'-ATAGATcgcCAGCAGACCCCACAGAAATTCACC-3' |
|  | **C1015W**  **V1018T** | **Fw** | 5'-GAAGATaccTATCTGCAAGAAACCAAAGGTGGTCG-3' |
|  |  | **Rv** | 5'-CAGATAggtATCTTCccaGGCTTTCTGAATATTGC-3' |
| **WP_106260381g2** | **T566V** | **Fw** | 5'-GAAGATgtgCATCTGCAGGATACCAAAGGTGGTC-3' |
|  |  | **Rv** | 5'-CAGATGcacATCTTCACAAACTTTCTGAACTTCCGG-3' |
|  | **A624I** | **Fw** | 5'-CTGCTGattATCTATCAGAATGCAACACCGCGTC-3' |
|  |  | **Rv** | 5'-ATAGATaatCAGCAGACCCCACAGGGTTG-3' |
| **AnPixJg2** | **W289C**  **T292V** | **Fw** | 5'-GAAGATgtcCACTTACAAGAAACCCAAGGTGGAC-3' |
|  |  | **Rv** | 5'-TAAGTGgacATCTTCgcaAACAGTCTTGATATCAGGG-3' |
|  | **A350I** | **Fw** | 5'-TTATTGattGCTTATCAAAACTCTGGTACCCGTGAG-3' |
|  |  | **Rv** | 5'-ATAAGCaatCAATAAACCCCATAATTTTTCCGCAGC-3' |

Mutation sites were denoted by small letters.
